# Creation of pure non-crystalline diamond nanostructures *via* room-temperature ion irradiation and subsequent thermal annealing

**DOI:** 10.1039/d1na00136a

**Published:** 2021-06-09

**Authors:** F. Picollo, A. Battiato, F. Bosia, F. Scaffidi Muta, P. Olivero, V. Rigato, S. Rubanov

**Affiliations:** Physics Department and “NIS Inter-departmental Centre”, University of Torino Torino 10125 Italy paolo.olivero@unito.it; National Institute of Nuclear Physics, Section of Torino Torino 10125 Italy; Applied Science and Technology Department, Politecnico di Torino Torino 10129 Italy; National Institute of Nuclear Physics, National Laboratories of Legnaro Legnaro 35020 Italy; Ian Holmes Imaging Centre, Bio21 Institute, University of Melbourne Victoria 3010 Australia

## Abstract

Carbon exhibits a remarkable range of structural forms, due to the availability of sp^3^, sp^2^ and sp^1^ chemical bonds. Contrarily to other group IV elements such as silicon and germanium, the formation of an amorphous phase based exclusively on sp^3^ bonds is extremely challenging due to the strongly favored formation of graphitic-like structures at room temperature and pressure. As such, the formation of a fully sp^3^-bonded carbon phase requires an extremely careful (and largely unexplored) definition of the pressure and temperature across the phase diagram. Here, we report on the possibility of creating full-sp^3^ amorphous nanostructures within the bulk crystal of diamond with room-temperature ion-beam irradiation, followed by an annealing process that does not involve the application of any external mechanical pressure. As confirmed by numerical simulations, the (previously unreported) radiation-damage-induced formation of an amorphous sp^2^-free phase in diamond is determined by the buildup of extremely high internal stresses from the surrounding lattice, which (in the case of nanometer-scale regions) fully prevent the graphitization process. Besides the relevance of understanding the formation of exotic carbon phases, the use of focused/collimated ion beams discloses appealing perspectives for the direct fabrication of such nanostructures in complex three-dimensional geometries.

## Abstract 2

It is possible to create full-sp^3^ amorphous nanostructures within the bulk crystal of diamond with room-temperature ion-beam irradiation, followed by an annealing process that does not involve the application of any external mechanical pressure.

## Graphical abstract

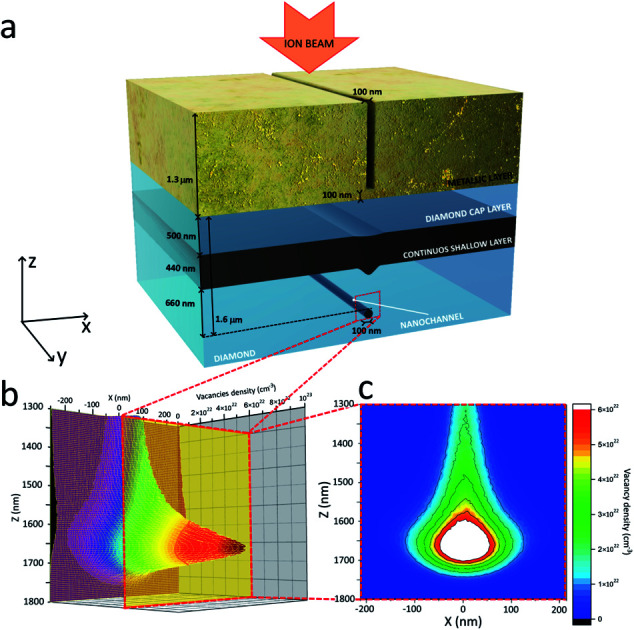

## 1. Introduction

Carbon is an extremely “versatile” chemical element due to the availability of different types of hybridized chemical bonds (sp^1^, sp^2^ and sp^3^), that determine a remarkable range of possible allotropic forms, both in bulk form and as nanostructures.^1^ In many respects, diamond lies at the very extreme of such a range, as far as bulk structures are concerned: due to is strong covalent sp^3^ bond, the diamond crystal is characterized by extreme mechanical (high hardness, low friction coefficient), optical (broad transparency from the near UV to the far IR), thermal (large thermal conductivity, low thermal expansion coefficient) and electrical (extreme dielectric strength, high carrier mobility) properties.^2^ These unique characteristics have motivated a remarkable body of scientific work aimed at better understanding its fundamental properties, as well as its synthesis and application in many different technological fields, ranging from high-power to quantum devices, encompassing biosensors, MEMS technology and much more.^3–5^ Not only has the systematic production of high-quality artificial diamond crystals *via* high-pressure-high-temperature (HPHT)^6^ and chemical-vapor-deposition (CVD)^7^ techniques made remarkable progress in the past decades, but the development of devices based on micro- and nano-crystalline diamond has also attracted ever-increasing interest, thanks to the fact that several appealing characteristics (most remarkably mechanical ones) are largely preserved in a material platform requiring less sophisticated synthesis methods.^8^

Moving towards more “defective” and technologically viable forms of sp^3^-bonded carbon, different forms of polycrystalline diamond,^9^ ultra-nanocrystalline diamond,^10^ nano-twinned diamond^11^ and amorphous diamond-like carbon^12,13^ have been widely investigated for several decades, with the promise of further expanding the applicability of extreme physical properties into technological landscapes in which synthesis and fabrication techniques can be realistically scaled to large production volumes. In this context, the higher thermodynamical stability of sp^2^-bonded carbon at room pressure and temperature conditions represents a fundamental limitation: in these conditions, graphite and graphite-like phases constitute the ultimate “ground state” for carbon structures when a critical amount of structural disorder is introduced. For this reason, substantial efforts have been made in the synthesis of amorphous carbon phases characterized by a high fraction of sp^3^ bonds,^14–16^ but the pursuit of a 100% fully sp^3^-bond amorphous carbon phase is still ongoing. A careful control of environmental parameters (pressure in particular) allows the engineering of novel forms of carbon, as demonstrated by the fact that exerting high (*i.e.* ∼10^2^ GPa) pressures on glassy carbon (*i.e.* an amorphous sp^2^ phase) yields the formation of phases characterized by high sp^3^ content with no long-range ordering, whose structural stability can to some extent be tuned if an equally careful control of temperature variable can be achieved.^17–19^ In this context, a powerful and versatile tool is represented by the local laser heating of different types of carbon structures under different mechanical stress conditions, either exerted from external pressure sources^20^ or established within the sample by the coexistence of carbon phases characterized by different densities and mechanical properties.^21^

Local laser heating was combined with the possibility offered by MeV ion irradiation to create sub-superficial graphitic structures within bulk diamond thanks to the strongly non-linear damage profile of energetic ions in matter. The ion-damage-induced collapse into a graphitic phase of layers with sub-μm thickness localized within the diamond crystal determines substantial local variations in both atomic density and mechanical parameters (Young's and shear moduli), that can in turn develop strong (*i.e.* ∼10 GPa) and highly localized internal stresses, without the need of using external pressure sources.^22^ In these conditions, optical absorption of the laser light at different power densities from the sub-superficial compressed graphitic layers allowed a fine control of local temperature variations, and thus an accurate exploration of the graphite–diamond–liquid triple point.^23^ In this context, the employment of other types of radiation (*e.g.* X-ray nano-beams) could be successfully employed to engineer structural damage with high spatial resolution, as already successfully demonstrated in other types of substrates.^24–26^

More recently, a careful control of the *in situ* laser-induced heating of glassy carbon kept at high (*i.e.* ∼50 GPa) pressure by means of a diamond anvil cell allowed the exploration of a very specific (and up to then scarcely studied) portion of the phase diagram of carbon, which resulted in the first demonstration of the synthesis of quenchable fully-sp^3^ bonded amorphous carbon phase. This stable amorphous phase of carbon was unequivocally demonstrated to be based on a sp^2^-free structure by means of high-resolution transmission electron microscopy (HRTEM) and electron energy loss spectroscopy (EELS), and exhibited properties of optical transparency, high density and extreme stiffness that were comparable to those of diamond.^20^

In the present work, we take advantage of a high-resolution lithographic technique based on the use of masked MeV ions to define sub-superficial amorphous nanostructures in the diamond bulk induced by atomic collisions. We demonstrate by means of HRTEM and EELS that these structures are lacking any measurable fraction of sp^2^ bonds, specifically because their size (*i.e.* ∼100–200 nm, depending upon fabrication parameters) and depth below the crystal surface (*i.e.* ∼1.6 μm) is such to inhibit any form of graphitization by the development of strong (*i.e.* >40 GPa) internal pressures. These results demonstrate for the first time the possibility of direct MeV-ion-beam writing with high spatial resolution a quenchable amorphous phase in diamond at room conditions, with no need of externally applied pressures.

## 2. Results and discussion

**Fig. 1:**
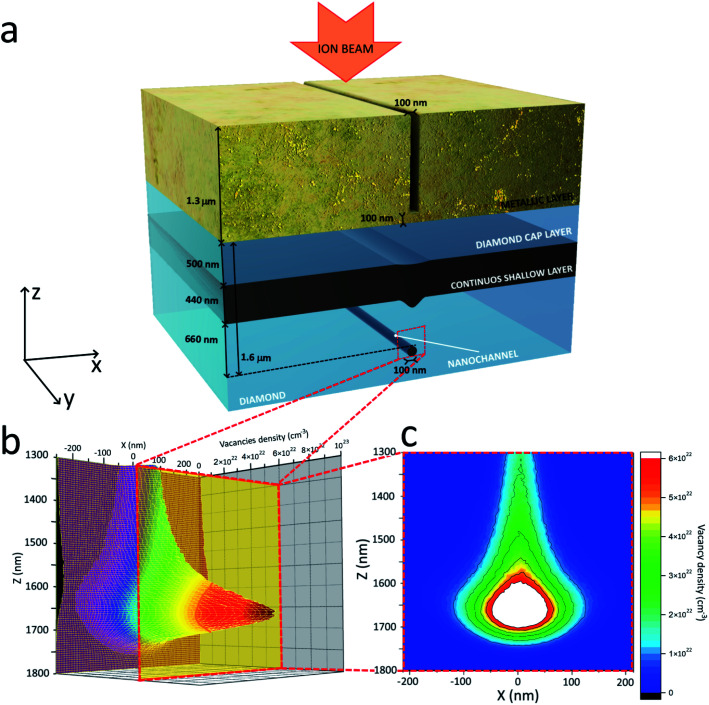
Sample geometry and profile of the ion-induced structural damage. (a) Schematic representation of the sample geometry: the metallic mask with the nanometric aperture determines the formation of both the continuous shallow layer and of the nanochannel upon MeV ion irradiation. (b) Three-dimensional plot of the cross-sectional profile of ion-induced damage density as resulting from SRIM simulation. (c) Corresponding two-dimensional plot: the size and shape of the region damaged beyond the estimated critical threshold (in red) corresponds to the features observed in Fig. 4a; note that the same plot is reported as an inset of Fig. 4a for sake of comparison with experimental data.

Radiation-hard contact masks were lithographically defined at high spatial resolution on single-crystal diamond samples, with the purpose of allowing ion irradiation across nanometer-sized regions. To achieve this, focused ion beam (FIB) micromachining was performed on a ∼1.3 μm thick copper layer deposited on the sample surface, resulting in the formation of linearly shaped nano-apertures with 100 nm lateral width, as schematically shown in Fig. 1. Notice that across the FIB-micromachined apertures the sample surface was not fully exposed, but rather a ∼100 nm thick metal layer was left at the bottom of the aperture to avoid the contamination of the diamond surface with the milling Ga^+^ ion beam. After mask preparation, the samples were irradiated with a 1 MeV He^+^ ion beam at 5 × 10^16^ cm^−2^ fluence. As schematically shown in the inset plot of Fig. 1, the energy and fluence of implanted ions was such that across the exposed areas a sub-superficial highly-damaged layer was formed in correspondence of the end-of-range “Bragg peak” of the ion damage profile, *i.e.* ∼1.6 μm. Conversely, the irradiation occurring just below the masked regions resulted in the formation of a shallow (*i.e.* ∼500 nm) damaged region. Overall, as schematically shown in Fig. 1a (and experimentally highlighted in Fig. 4a), ion-induced structural damage resulted in the formation of sub-superficial narrow regions (referred to as “nanochannels” in the following) located below an extended shallow region (referred as “continuous shallow layer” in the following). The ∼500 nm thick layer of diamond comprised between the continuous shallow layer and the sample surface will be referred as “cap layer” in the following. The mask thickness was specifically chosen to allow the formation of the continuous shallow layer, that acted as the “reference” damaged region with respect to the nanochannels. The main difference between these two types of structures consists in the depth at which they are formed, since they are created upon the same irradiation carried over the very same time.

After MeV ion irradiation and subsequent mask removal, the samples were thermally annealed in vacuum at 950 °C, with the scope of allowing the structural reorganization of the highly damaged buried nano-regions, while removing residual damage from the regions irradiated at intermediate depths.

The nanochannels of highly-damaged carbon phase are expected to form where the structural damage (here parameterized as a volume density of created vacancies, as predicted by the SRIM Monte Carlo simulation code^27^ in a linear approximation) exceeds a critical threshold, whose value has been estimated as ∼(6.4 ± 1.5) × 10^22^ cm^−3^ on the basis of the measured dimensions of the nanostructures (see Fig. 1b, c and 4a).

This value is in good agreement with previous estimations of the parameter, commonly referred to as “graphitization threshold”, in the (5 − 7) × 10^22^ cm^−3^ range.^28–32^ As shown in Fig. 1 (as well as in the inset of Fig. 4a), the SRIM-based model of the damage profile (which also suitably describes the trajectories of laterally straggled ions) accurately predicts not only the ∼1.6 μm depth of the nanochannels below the surface, but also their overall shape.

**Fig. 2:**
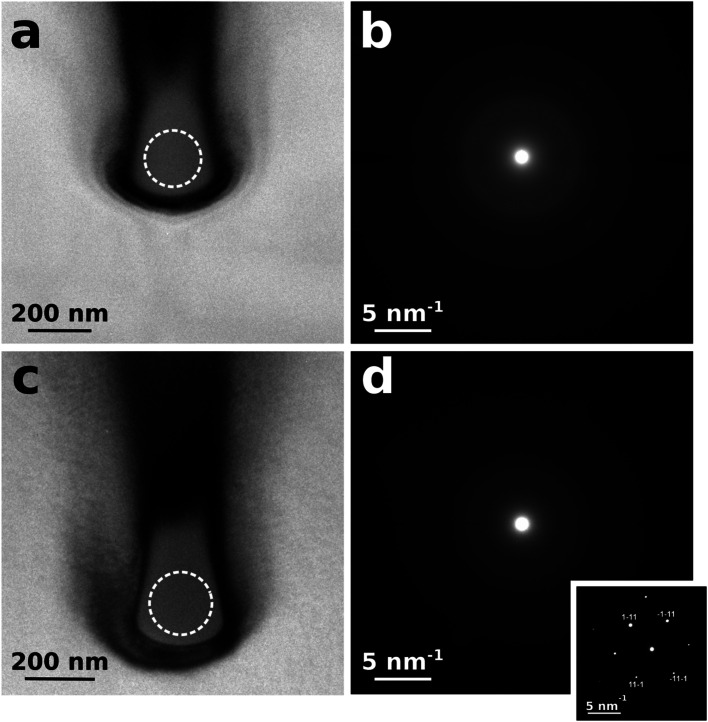
Results of cross-sectional TEM characterization of a nanostructure. (a) Bright-field TEM micrograph from the as-implanted structure and (b) corresponding diffraction pattern collected from the area highlighted by the dashed circle in (a). (c) Bright-field TEM micrograph from the same structure after thermal annealing and (d) corresponding diffraction pattern collected from the area highlighted by the dashed circle in (c). A diffraction pattern from the surrounding diamond matrix is reported in the inset for reference purpose.

As shown in Fig. 2a and b, the bright-field TEM cross-sectional micrograph and related selected area diffraction pattern indicate that the as-irradiated microstructures consist of a fully amorphized phase. Remarkably, the same is observed also after the annealing step (see Fig. 2c and d), thus indicating that the thermal process stabilizes the structures without inducing any re-crystallization of either sp^2^ or sp^3^ phases. Dark contours are also visible around the nanostructures, indicating lattice strains due to a high local concentration of point defects. The diffraction patterns in Fig. 2b and d only show broad rings that are typical of amorphous structures. It is worth remarking that the radius of the first ring correlates with the positions of the {111} diffraction spots generated from the surrounding crystalline diamond matrix, as reported in the reference diffraction pattern reported in the inset of Fig. 2. This confirms that the probed phase is fully amorphized. The absence in the diffraction patterns of features related to sp^2^ bonding (*i.e.* rings corresponding to the {002} lattice plane of graphite) can be attributed to a low fraction of sp^2^ bonds or to a predominant orientation of graphite basal plains normal to the electron beam direction.

**Fig. 3:**
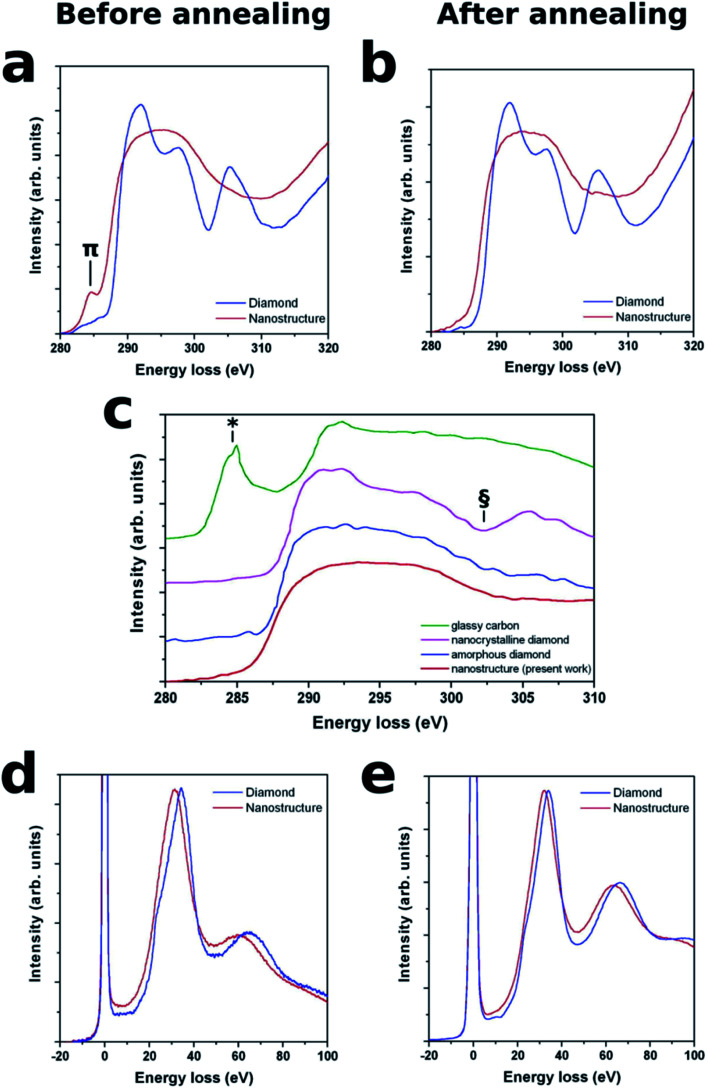
Results of cross-sectional EELS spectroscopy of the nanostructures. K-edge absorption features of both the nanostructure (red plots) and the surrounding diamond matrix (blue plots) are reported for both the as-implanted (a) and thermally processed (b) sample. The pre-edge peak at ∼285 eV, which is attributed to π-bonded carbon, is visible in a, while it is entirely absent in (b). (c) Present experimental data are compared to the data reported for fully-sp^3^ bonded amorphous carbon in ref. 20, as well as to the characteristic spectra of glassy carbon (in which the ∼285 eV feature is labeled as *) and nanocrystalline diamond (whose characteristic post-edge structure is labeled as §). Low-loss spectra exhibit a characteristic downshift in the plasmon-related features, both before (d) and after (e) thermal processing.

In order to provide direct insight into the nature of the chemical bonds within the amorphized nano-regions, EELS analysis was carried both before and after thermal annealing, in the energy ranges corresponding to the K absorption edge of carbon and the plasmonic energy loss. As far as the former energy range is concerned (see Fig. 3a), the K-edge EELS spectra acquired from the nanostructure before thermal annealing are entirely lacking the articulated post-edge structures observed in the corresponding spectra acquired from the surrounding diamond matrix. Remarkably, this clear distinction in EELS spectral features is fully preserved after the annealing step, thus indicating that no phases attributable to crystalline diamond can be detected upon thermal processing.

Note that the spectra acquired from the nanostructures before thermal treatment exhibit a well-defined (although not particularly intense) absorption pre-edge peak at ∼285 eV that is unequivocally attributed to π-bonded carbon,^33^ thus indicating that a fraction of sp^2^ bonds is indeed present in the as-implanted phase. Contrarily to what is commonly observed in amorphized carbon, this spectral feature does not increase upon thermal annealing, but rather completely disappears, which unequivocally indicates that the sp^2^ bonds are absent from the annealed nanostructure within the detection limit of this very sensitive technique.

For the sake of comparison, Fig. 3c reports our experimental data together with the EELS spectrum collected from the fully sp^3^-bonded amorphous carbon phase investigated in ref. 20: the mutual similarity is striking, particularly considering that both spectra entirely lack the features associated with glassy carbon and nanocrystalline diamond (marked as * and § in the respective reference spectra). The EELS features in the low-energy-loss range reported in Fig. 3d and e exhibit plasmon peaks which are indicative of the electron densities in the corresponding phases. Both before and after thermal annealing, a systematic shift to lower energy losses of the plasmon peak positions is observed from the nanosctructures with respect to the ∼34 eV peak, which is characteristic of the surrounding diamond matrix.^14,34–36^ These shifts can be interpreted on the basis of the lower atomic density of the nanostructures, by adopting the following formula:^36–38^
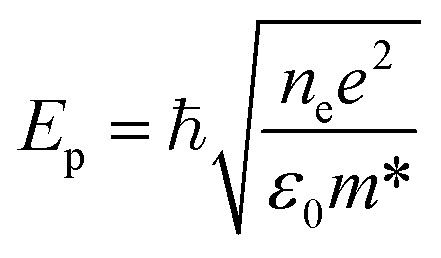
where *E*_p_ is the plasmon energy, ℏ is the Planck constant, m* and *e* are the effective mass and charge of electron, *ε*_0_ is the vacuum dielectric constant and *n*_e_ is the valence electron density.

Given the mass density (*i.e.* 3.515 g cm^−3^) and plasmon peak position (*i.e.* ∼34 eV) of the surrounding diamond matrix, and under the assumption that the same electron effective mass can be adopted for the different phases under investigation,^38^ it is possible to estimate the electron and (and thus mass) density within the nanochannels from the position of the corresponding plasmon peaks. Under these approximations, the (31.0 ± 0.3) eV and (32.6 ± 0.3) eV plasmon peak positions measured from the nanostructures before and after thermal annealing yield mass density estimations of (2.92 ± 0.06) g cm^−3^ and (3.27 ± 0.07) g cm^−3^, respectively.

This result is indicative of the fact that: (i) the implantation process results in a substantial density variation within the nanochannels, despite the strong compressive stress exerted by the rigid surrounding diamond matrix; and (ii) the disappearing of sp^2^ bonds within the nanochannels upon thermal annealing determines a substantial increase of mass density with respect to the as-implanted sample, which closely approaches the density of pristine diamond and is fully compatible with the estimation (*i.e.* 3.3 g cm^−3^) provided for the fully sp^3^-bonded amorphous carbon phase reported in ref. 20.

**Fig. 4:**
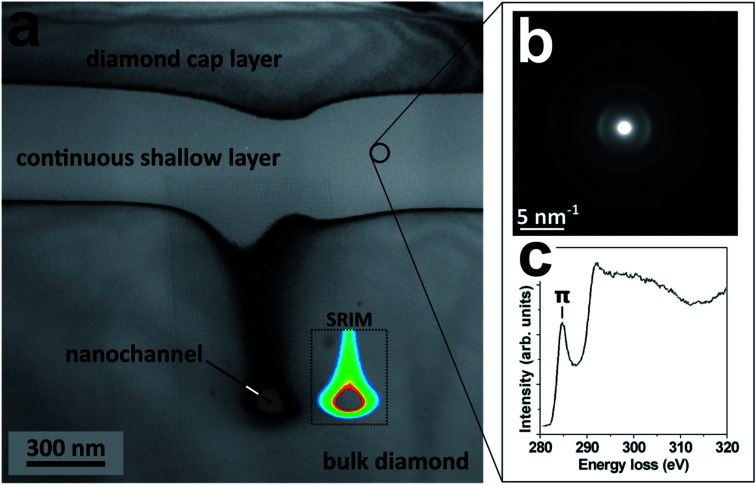
Structural properties of the continuous shallow layer located above the nanostructures, following thermal annealing. (a) Cross sectional bright-field TEM micrograph from the annealed structure: the labels indicate the diamond cap layer, the continuous shallow layer and the nanochannel embedded in the bulk diamond; the inset labeled by “SRIM” reports the two-dimensional damage density plot reported in Fig. 1c, for sake of comparison. (b) TEM diffraction pattern collected from a random region of the continuous shallow layer. (c) Corresponding EELS spectrum, clearly exhibiting the strong absorption pre-edge peak at ∼285 eV which is indicative of a large fraction of sp^2^ bonds.

Finally, we remark that thermal annealing results in radically different structural features across the previously defined “continuous shallow layer” located above the nanochannels (see Fig. 4a). While (as much as observed from the nanochannels) the continuous shallow layer is characterized by TEM diffractometry features that are indicative of a fully amorphized phase (see Fig. 4b), the EELS spectrum (see Fig. 4c) exhibits the strong absorption pre-edge peak at ∼285 eV. Such a difference is attributed to the different geometries of the two types of structures, while all other fabrication and processing parameters (irradiation, thermal annealing) are the same. This strongly indicates that the peculiar stress field developed in correspondence of the nanostructures is primarily responsible for the formation of an amorphous full-sp^3^ network.

Our interpretation is confirmed by 2D finite element method (FEM) mechanical analysis. By simulating the constrained expansion undergone by the two implanted regions, it is possible to highlight a significant volumetric stress build up in the 40–50 GPa range (consistently with results in ref. 20) upon ion implantation.

**Fig. 5:**
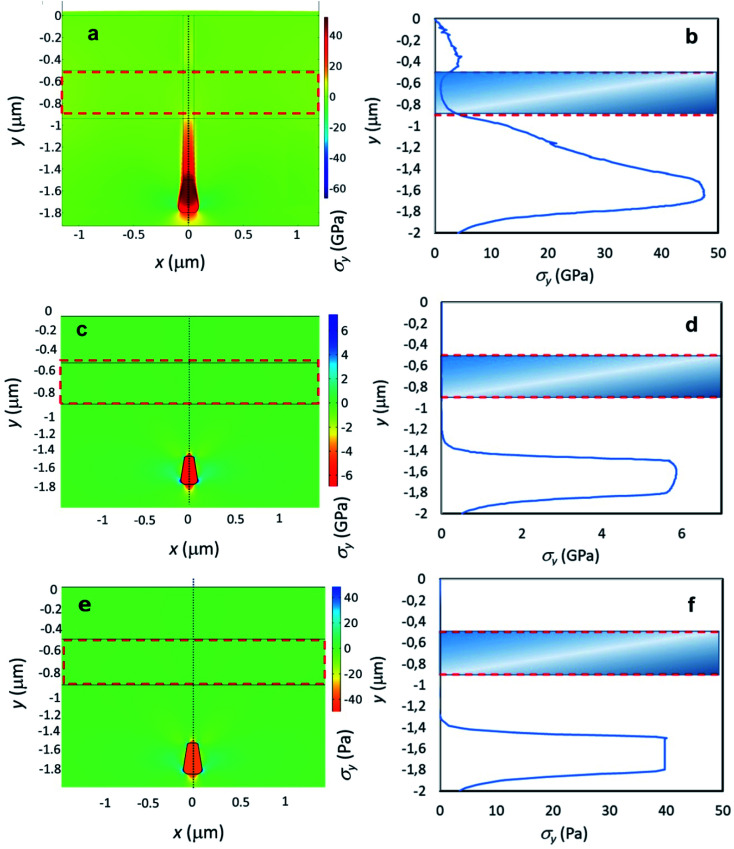
Results of FEM simulations of stress distributions across the structures. (a) Map of the principal stress in the vertical direction (*σ*_*y*_) in a cross section of the as-implanted diamond: stresses develop in the nanochannel, and are negligible in the shallow layer (dashed line). (b) *σ*_*y*_ variation along the dotted line in (a); the shaded region indicates the shallow layer location. (c and d) Results of analogous simulations carried for the nanostructure after thermal annealing, under the assumption that this process results in a conversion to a graphitic phase: residual stresses in the GPa range are still persistent. (e and f) Results of analogous simulations carried for the nanostructure after thermal annealing, under the assumption that the latter process results in a conversion to a fully sp^3^-bonded amorphous carbon phase: very small residual stresses in the 10 Pa range are obtained in this case.

However, as shown in Fig. 5a, the shallow layer (region encircled by the dashed line) does not undergo significant stresses in the vertical (*y*) direction, while the latter tends to accumulate in the end-of-range region of the nanochannel, due to the confining effect of the surrounding pristine diamond material. The depth variation of *σ*_*y*_ stresses in the nanochannel (Fig. 5b), which reach a peak value of about 48 GPa, while remaining negligible in the shallow layer (shaded area), are identified as the factors that are responsible for the different structures observed in the two regions. Fig. 5c and d show the results of analogous numerical simulations carried for the nanostructure after thermal annealing, under the assumption that the latter process results in a conversion to a graphitic phase. In this case, a distribution of relatively high compressive stresses (in the GPa range) still persists. It is worth remarking that stress fields of this order of magnitude are indeed observed in graphitized microstructures created with this technique, such as the ones reported in ref. 22. The experimental observation that thermal annealing does not result in the graphitization of the nanostructure is therefore attributed to the fact that the strong stress fields established around the nanostructure upon ion implantation are instead fully relaxed (*i.e.* residual stresses in the Pa range) upon the formation of a fully sp^3^-bonded amorphous carbon phase, as confirmed by the simulations reported in Fig. 5e and f. In our interpretation, the stress state responsible for the transition to sp^3^ bonds is established before the thermal annealing. The initially strongly stressed nanochannels therefore transition to a fully sp^3^-bonded amorphous phase upon thermal annealing, and only subsequently are the stresses relaxed.

As mentioned above, an experimental assessment of the local stresses established across amorphized/graphitized structures embedded in the diamond matrix is in general possible by means of confocal micro-Raman spectroscopy, both before and after thermal annealing, as already demonstrated for micrometer-sized regions.^22^ This was not possible (either by conventional or tip-enhanced Raman spectroscopy) in the case of the nanostructures reported in the present work, due to (respectively) limited spatial resolution and spectral sensitivity of the available techniques. Nonetheless, it is worth remarking that EELS provided direct experimental insight into the local mass density of the nanostructures, which directly translated into the above-described numerical simulations.

## 3. Conclusions

In conclusion, the reported results indicate the possibility of creating an amorphous carbon phase entirely based on sp^3^ chemical bonds, by means of ion-induced structural damage in nanometer-sized regions embedded within the bulk diamond structure, followed by thermal annealing. As confirmed by numerical simulations, the strong three-dimensional mechanical stress state developed within the nanostructures is the required condition for the formation of this peculiar carbon phase without the need of applying external pressure during the annealing process: the key role played in the formation of this phase by internal stress fields surrounding the nanostructures is demonstrated by the fact that control structures with different geometries, and hence stress states, produce a radically different amorphous phase containing a substantial fraction of sp^2^ bonds. It is worth remarking that, although playing an essential role, the high mechanical stresses established across the nanostructures are not sufficient for the formation of the reported fully sp^3^-bonded amorphous phase. As reported in Fig. 3a, a fraction of sp^2^-bonded carbon is indeed persistent in the strongly stressed nanostructures formed upon ion implantation, and it is converted only upon the subsequent thermal annealing. The necessity of carefully controlling both pressure and temperature parameters for the formation of the reported carbon phase is qualitatively consistent with what is reported in previous works^17–19^ and particularly in ref. 20. Furthermore, it is coherent with our current understanding of the complex pressure temperature landscape around the triple point in the phase diagram of carbon.^23^ We envisage that an accurate numerical simulation of this carbon-based system in the reported range of pressure/temperature parameters would shed significant insight into the mechanisms leading to the formation of the fully sp^3^-bonded amorphous carbon phase.

The results presented in this work provide important information regarding the mechanisms leading to the formation of fully-sp^3^-based amorphous phases in carbon, and display appealing applications in fields where high-pressure carbonaceous phases could be implemented in integrated devices, such as for example room-temperature superconducting devices.^39^

Besides the fundamental relevance of this finding in the understanding of the formation of exotic carbon phases, the use of focused/collimated ion beams enables the direct fabrication of such nanostructures in complex patterns and arrangements, with appealing perspectives in nanomechanical systems and integrated nano-optics.

## 4. Methods

### 4.1 Samples

The experiments were performed with equivalent results on different types of commercially-available artificial single-crystal diamond samples, namely a 3 × 3 × 0.5 mm^3^ sample produced by Element Six™ with the chemical vapor deposition technique, and a 3 × 3 × 1.5 mm^3^ sample produced by Sumitomo Electrics™ with the high pressure high temperature synthesis technique. The former sample is classified as a type IIa “optical grade” crystal, with substitutional nitrogen and boron concentrations lower than 1 ppm and 0.05 ppm, respectively. The latter sample is classified as a type Ib crystal, with substitutional nitrogen concentration comprised between 10 ppm and 100 ppm. In both cases, the samples are cut along the 100 crystal direction and are optically polished on the two opposite large faces. After accurate surface cleaning, a ∼1.3 μm thick Cu layer was deposited by thermal evaporation in high vacuum conditions. Conventional FIB micromachining with a 30 keV Ga^+^ ion beam was used for the mask fabrication. A Quanta 3D FEG DualBeam™ apparatus equipped with Nanometer Pattern Generation System (from J. C. Nabity) was used for the patterning of linear nano-apertures of 100 nm width. A protective thin layer (∼100 nm) of the mask was left in correspondence of each aperture, in order to avoid the superficial damage induced by Ga^+^ ions. After the FIB mask definition, the sample was irradiated with a beam of 1 MeV He^+^ ions at the 0° beam line of the AN2000 accelerator of the INFN National Laboratories of Legnaro. Ion current density was ∼500 nA mm^−2^ and the irradiation fluence was 5 × 10^16^ cm^−2^. After mask removal, thermal annealing was carried at 950 °C for 2 hours in vaccum with a slow (*i.e.* 5 °C min^−1^) heaing and cooling rate.

### 4.2 Sample characterization

Sample characterization was performed at the Ian Holmes Imaging Centre of the Bio21 Institute (University of Melbourne). 150 nm-thick cross-sectional lamellae were cut in the {100} crystallographic direction by conventional FIB micromachining employing 30 keV Ga^+^ ions. In order to reduce FIB-induced damage layers, the lamellae were finally cut with a 5 keV Ga^+^ beam from the same FIB facility. High-resolution transmission electron microscopy (HRTEM) were performed with a Tecnai TF20 electron microscope (S-TWIN objective lens, 0.24 nm point resolution) transmission electron microscope operated at 200 keV. Selected area diffraction patterns (SADP) were collected with smallest aperture (∼180 nm effective diameter in specimen plane). Electron energy loss spectroscopy (EELS) measurements and spectrum imaging were conducted in STEM mode, with 1 nm probe beam diameter, 2.2 mrad convergence semi-angle and 16 mrad collection semi-angle at 200 kV (FEI Tecnai TF30) using Gatan GIF Quantum™ 965 energy filter with dual EELS capability. All TEM and EELS data processing was carried out using Gatan Digital Micrograph (DM) software.

### 4.3 Numerical simulations

The MeV ion induced structural damage profiles were numerically modeled by means of the SRIM Monte Carlo Code (2013.00 version).^27^ All simulations were performed in the “Detailed calculation with full damage cascades” mode, by setting a displacement energy value of 50 eV for diamond.^40,41^ The beam was assumed to be impinging on the surface at normal incidence with the mask perfectly aligned to the ion beam. A large (*i.e.* >100 000) number of ion trajectories was simulated. The SRIM output yields profiles of linear damage density, parameterized as number of vacancies per unit length in the depth direction. Volumetric vacancy densities were estimated from the above-said linear density profiles and the implantation fluences, by modeling the cumulative effect of implanted ions within a simple linear approximation, which does not take into account complex processes such as self-annealing, ballistic annealing and defect interaction.

To gain further insight in the process of amorphization and estimate the stresses acting on the irradiated region, 2-D FEM simulations were performed using Comsol Multiphysics. Consistently with the results of cross-sectional TEM microcopy (see Fig. 4a), a 5 × 5 μm^2^ diamond cross section was considered, in which a 0.1 × 1.8 μm^2^ implanted diamond strip is incorporated, representing the nanochannel, including a terminal trapezoidal region to account for straggling effects, and a 5 × 0.5 μm^2^ strip representing the shallow layer (see Fig. 5). For the as-implanted sample, material parameters are: diamond density *ρ*_d_ = 3.52 g cm^−3^, amorphous carbon density *ρ*_aC_ = 2.06 g cm^−3^, diamond Young's modulus *E*_d_ = 1220 GPa, amorphous carbon Young's modulus *E*_aC_ = 21.38 GPa.^42,43^ The density of the implanted region before annealing was calculated as a function of the induced damage density as reported in ref. 44, *i.e.*:*ρ*(*y*) = *ρ*_d_ − (*ρ*_d_ − *ρ*_aC_)*Φ*(*y*)
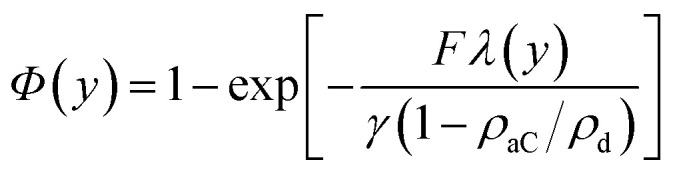
where *γ* = 1.77 × 10^23^ cm^−3^ is the atomic density of diamond, *F* is implantation fluence and *λ*(*y*) is the linear vacancy density, as derived from SRIM code. The equation accounts for defect recombination and damage saturation effects occurring in the crystal.^45^

The Young's modulus decrease as a function of the vacancy density can be expressed as:*E*(*y*) = *E*_d_ − (*E*_d_ − *E*_aC_)*Φ*(*y*)

The density decrease due to irradiation generates a constrained expansion of the implanted volume, *i.e.* residual strains in the *i* = *x*,*y* directions that can be expressed as follows:
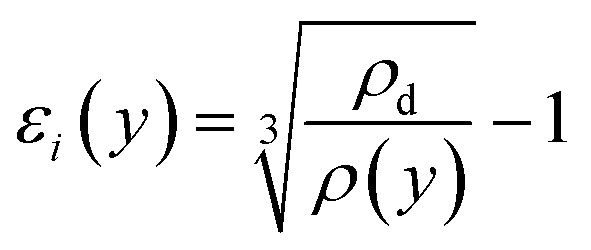


In the case of the sample after thermal annealing, the simulations were carried under two alternative hypotheses, *i.e.* a conversion of the nanostructure to a graphitic phase (see Fig. 5c and d) or to a fully sp^3^-bonded amorphous carbon phase (see Fig. 5e and f). In the former case, the following structural parameters were adopted: *ρ*_g_ = 2.1 g cm^3^, Young's modulus *E*_g_ = 21 GPa. In the latter case, the following structural parameters were adopted: *ρ*_a–d_ = 3.3 g cm^3^ [ref. 20, this work], *E*_a–d_ = 1123 GPa.^20^

## Author contributions

F. P. conceived this study and coordinated the experimental campaign. A. B. and F. S. M. prepared the samples (masking and post-irradiation processing). V. R. and F. P. prepared the samples (ion irradiation). F. B. developed the numerical model of the internal stresses. P. O. contributed to data analysis and compiled the manuscript (with contributions from all co-authors). S. R. performed TEM and EELS characterization.

## Conflicts of interest

There are no conflicts to declare.

## Supplementary Material
